# Mother–Infant Physical Contact Predicts Responsive Feeding among U.S. Breastfeeding Mothers

**DOI:** 10.3390/nu10091251

**Published:** 2018-09-06

**Authors:** Emily E. Little, Cristine H. Legare, Leslie J. Carver

**Affiliations:** 1 Department of Psychology, University of California, San Diego, CA 92093, USA; ljcarver@ucsd.edu; 2 Department of Psychology, The University of Texas at Austin, TX 78712, USA; legare@austin.utexas.edu

**Keywords:** responsive feeding, breastfeeding, breastmilk, babywearing, co-sleeping, mother–infant interaction, feeding cues, maternal responsiveness, mother–infant physical contact, proximal care

## Abstract

Responsive feeding—initiating feeding in response to early hunger cues—supports the physiology of lactation and the development of infant feeding abilities, yet there is a dearth of research examining what predicts responsive feeding. In non-Western proximal care cultures, there is an association between responsive feeding and mother–infant physical contact, but this has not been investigated within Western populations. In two studies, we tested whether mother–infant physical contact predicted feeding in response to early hunger cues versus feeding on a schedule or after signs of distress among U.S. breastfeeding mothers. With an online questionnaire in Study 1 (*n* = 626), physical contact with infants (via co-sleeping and babywearing) predicted increased likelihood of self-reported responsive feeding. Mothers who reported responsive feeding were more likely to exclusively breastfeed for the first six months, breastfeed more frequently throughout the day, and had a longer planned breastfeeding duration than mothers who reported feeding on a schedule or after signs of infant distress. In Study 2 (*n* = 96), a three-day feeding log showed that mother–infant physical contact predicted feeding in response to early hunger cues but mother–infant proximity (without physical contact) did not. In sum, our results demonstrate that physical contact with infants may shape breastfeeding behavior among U.S. mothers, highlighting a connection between social interaction and infant nutrition that warrants further investigation.

## 1. Introduction

Breastfeeding is internationally recognized as the optimal nutrition for infant health and development [1,2], yet most U.S. mothers do not meet the World Health Organization’s recommendation of exclusive breastfeeding for six months and continued breastfeeding for 24 months and beyond [3,4,5]. Responsive feeding—initiating feeding in response to early hunger cues such as lip smacking and bringing hands to mouth—decreases breastfeeding challenges by supporting the physiology of lactation and the development of infant feeding abilities [6,7,8]. Most mothers in the U.S. report crying as the primary reason for initiating feeding, which is an indication of infant distress rather than an early cue for hunger [9]. Ethnographic accounts of infant care report high levels of responsive feeding among mothers in proximal care cultures in which infants are in near-constant physical contact with mothers [10,11]. No research to date has systematically documented the association between mother–infant physical contact and responsive feeding among U.S. mothers. Here, we use convergent methods—an online questionnaire and an at-home feeding log—to examine whether mother–infant physical contact facilitates increased responsive feeding among U.S. breastfeeding mothers.

Ethnographic accounts of infant care in non-Western cultures show that responsive feeding is associated with proximal caretaking practices, a style of parenting characterized by mother–infant physical contact through the day and night. For example, Konner and colleagues note that !Kung San caregivers of Northwestern Botswana are in near-constant physical contact with infants and respond to their needs quickly [12]. Hewlett and colleagues have noted similar practices among the Aka foragers of Central Africa, who also keep infants close throughout the majority of the day and respond promptly to signs of distress [13]. Responsiveness in this context of physical closeness often manifests in the form of offering the breast for nursing [14,15,16]. Breastfeeding promptly in response to early hunger cues may preclude the need for infants to display overt signs of distress [17,18]. When in sustained body contact, mothers can sense infants’ needs via subtle physical movements and do not wait to see or hear overt signs of discomfort [19,20]. This leads to high frequency of breastfeeding in proximal care cultures, up to several times per hour [21,22]. Mothers also show acute awareness of subtle elimination signals, demonstrated by moving infants into an appropriate position immediately before infants empty their bowels [23].

A limitation of the ethnographic literature is that the connection between high levels of physical contact and increased maternal responsiveness is embedded within the broader parenting profile of proximal care, comprising a distinct set of parenting beliefs that may also be driving increased responsiveness. These beliefs are referred to as parental ethnotheories, or cultural parenting models used to define parental roles and goals for children [24]. It is an open question whether the mother–infant physical contact characteristic of proximal care facilitates increased responsiveness or whether the increased responsiveness is simply part of the psychological model of socialization goals and parenting beliefs.

The connection between mother–infant physical contact and maternal responsiveness has not been investigated outside of the proximal care context of small-scale, indigenous communities. Infant care among middle-class Euro-American parents in the U.S. is described as distal care, which is characterized by face-to-face interaction and object stimulation [25]. Yet a movement within many Western countries to adopt philosophies of “natural parenting” and “attachment parenting” has motivated some parents to adopt a parenting style that resembles proximal care, emphasizing high levels of physical contact and extended breastfeeding. There is a dearth of information about these practices in Western culture. One study reported that parents in London who identify with proximal care parenting philosophies had over 50% more physical contact with their infant than parents practicing distal care, which resulted in the proximal care infants crying 50% less and breastfeeding for longer [26]. The extent to which the practices of proximal care—including babywearing (carrying infants for extended periods on the body using a sling or wrap) and co-sleeping (bed-sharing with infants to maintain physical contact throughout the night)—predict increased maternal responsiveness during feeding among U.S. mothers is currently unknown.

Our objective in the current investigation was to test whether mother–infant physical contact predicts variation in responsive feeding among U.S. breastfeeding mothers. Though very little is known about the predictors of responsive feeding among U.S. mothers, mothers who breastfeed directly from the breast—in comparison with bottle-feeding—are more likely to be responsive to early hunger cues [27] and are also more likely to breastfeed for a longer duration [28,29]. This disparity in responsive feeding between direct breast- versus bottle-feeding has been explained by several different factors, including the salient visual cue of the emptying bottle, prompting mothers to use quantity consumed to guide feeding rather than infant hunger and fullness cues [30]. Another potential explanation is the increased maternal sensitivity promoted by the oxytocin release during skin-to-skin contact [31]. We examined predictors of responsive feeding solely among mothers feeding directly from the breast.

In two studies, we used convergent evidence—a self-report questionnaire (Study 1) and an at-home feeding log (Study 2)—to test the hypothesis that mother–infant physical contact predicts responsiveness to infant hunger cues among U.S. breastfeeding mothers. Whereas questionnaires can assess overall reported responsiveness or philosophies about feeding, evidence can be strengthened if it is combined with live documentation of each feeding session, allowing us to capture not only variation between individuals in feeding strategies but also variation within individuals in their likelihood of responding to early hunger cues. Cross-cultural variation in mother–infant physical contact is associated with a set of socialization goals characteristic of proximal care culture, and even subtle differences in beliefs and intentions regarding breastfeeding can affect breastfeeding behavior [32]. We therefore used Keller’s [33] parental ethnotheories questionnaire to assess maternal beliefs about breastfeeding and responsiveness in Studies 1 and 2. We hypothesized that mother–infant physical contact would be associated with increased maternal responsiveness to infant hunger cues in the context of breastfeeding.

## 2. Study 1

Responsive feeding supports the supply and demand physiology of lactation and works in accordance with the range of individual variation in infant feeding needs [34]. It may also help protect against perceived insufficient milk syndrome, one of the primary reasons mothers end breastfeeding earlier than planned [35]. Though many lactation education programs now recommend responsive feeding as best practice for successful breastfeeding [36], alternate recommendations also exist. For example, starting in the 18th century, European male pediatricians recommended that infants be fed on a strictly regulated schedule [37], a practice which is still promoted today, both informally by parenting blogs and in certain health care settings. Waiting for crying and feeding on a set schedule are both problematic, because they neglect the ability of infants to communicate their hunger, creating a mismatch between infant hunger and feeding time. This mismatch has been linked to problems with early self-regulation and childhood obesity [38,39,40], in addition to breastfeeding-specific problems of latching difficulties and perceived insufficient milk supply, all of which may contribute to ending breastfeeding earlier than recommended.

Despite the known consequences of not practicing responsive feeding, there is a dearth of information regarding what individual level factors predict responsive feeding, especially among middle-class Euro-American mothers. Study 1 addressed two research question. First, do behaviors consistent with proximal care (i.e., babywearing, co-sleeping) predict a responsive breastfeeding philosophy? We hypothesized that mothers who practiced high level of physical contact through the day (via babywearing) and through the night (via co-sleeping) would be more likely to report a responsive feeding philosophy. Second, does having a responsive feeding philosophy predict improved breastfeeding outcomes? We predicted that self-reported responsive feeding would be associated with an increased likelihood of exclusive breastfeeding for the first six months, increased feeding frequency, and longer planned breastfeeding duration.

### 2.1. Materials and Methods

This study was conducted in accordance with the Declaration of Helsinki, and the protocol was approved by the Institutional Review Board of University of California, San Diego (protocol number 130567 “Culture and Infant-Caregiver Interactions”). We recruited mothers (*n* = 626) of newborn to 24-month-old infants to fill out an online questionnaire. These dyads were recruited from social media postings within U.S.-based parenting groups. After mothers expressed interest in participating in the study, they were contacted electronically by a research assistant who explained the protocol and obtained consent. Participants filled out the anonymous online questionnaire from their home.

Demographic information for the sample is included in Table 1.

The online questionnaire (administered through Google Forms) assessed demographic factors; parenting practices that facilitate mother–infant physical contact (babywearing, co-sleeping); maternal beliefs; and infant feeding philosophies, practices, and outcomes. We collected basic sociodemographic information from all mothers and controlled for these factors in each statistical model, including infant age (in months), maternal education level (high school, college, or graduate degree), current employment status (at home not working, working outside of the home), and hours per week that the child spends in daycare. These factors were chosen because past research has indicated these variables may be important for predicting breastfeeding behavior [41], and they play a role in how much time the mother spends with her child, potentially impacting ability to recognize or respond to feeding cues.

To assess mother–infant physical contact during the day, mothers were asked about infant carrying practices: “What is the primary method you use to transport your baby?” with the following response options: babywearing or other (i.e., “arms”, “stroller/seat”). To assess nighttime physical contact, mothers were asked “Where does your baby currently sleep?” with the following response categories: co-sleeping (“In the same bed as me”) or mixed/other (“in the same room, but a separate bed”, “in a separate room”, or “mixed”).

Maternal beliefs about responsiveness were assessed with Keller’s 10-question parental ethnotheory questionnaire that solicits degree of agreement with parenting statements regarding the care of a three-month-old infant. Responses to each item were on a scale from one (completely disagree) to five (completely agree). Responses from each participant were compiled to form a proximal care belief score, calculated by summing responses from all questions aimed at measuring alignment with goals of proximal care parenting culture then subtracting the sum of responses to all questions designed to test alignment with goals of distal care parenting culture. The range of possible scores was negative 20 to positive 20. Positive scores indicated that mothers were more aligned with the values of proximal care culture than distal care culture, and a higher score indicated a greater agreement with the parenting goals characteristic of proximal care culture.

To assess feeding philosophy, each mother was asked to choose the option that best described her feeding strategy with the options: responsive (“on demand”) or schedule/mixed (“feeding schedule”, “mix of both”). To assess breastfeeding outcomes, mothers of infants six months of age and younger were asked about exclusive breastfeeding (only breastmilk, as recommended by the World Health Organization and other international health organizations for the first six months of life) versus non-exclusive breastfeeding (supplementing breastmilk with formula, solids, or other liquids). Breastfeeding frequency throughout the day was assessed by asking mothers how many times per day they usually breastfeed their child (number). We assessed planned breastfeeding duration by asking mothers how many months they planned to breastfeed their child for (number in months).

In our analyses, we first sought to describe the beliefs and practices of U.S. breastfeeding mothers. We then examined the degree to which engagement in the beliefs and practices of proximal care predicted responsive feeding and whether responsive feeding predicted breastfeeding behavior. To examine whether proximal care beliefs (proximal care belief score) and practices (babywearing, co-sleeping) predicted self-reported responsive breastfeeding, we conducted multistep logistic regressions with feeding philosophy (responsive, scheduled/mixed) as the outcome measures and proximal care beliefs (proximal care beliefs score) and practices (babywearing, co-sleeping, or both) as the predictor measures, controlling for infant age, maternal education and employment, and hours per week that the child spends in daycare.

To examine whether reporting a responsive feeding philosophy predicted improved breastfeeding outcomes, we conducted separate logistic regressions with feeding strategy (responsive, scheduled/mixed) as the predictor measure—controlling for infant age, maternal education, maternal employment, and hours per week that the child spent in daycare—and exclusive breastfeeding (yes versus no) as the outcome measure (for mothers of infants six months of age and younger, *n* = 217). Controlling for the same demographic variables, we conducted linear regressions with breastfeeding frequency (number of times per day) and planned duration of breastfeeding (in months) as continuous outcome measures.

### 2.2. Results

All descriptive statistics for Study 1 are included in Table 2.

We first tested proximal care predictors of responsive breastfeeding. In Step 1 of the model, controlling for infant age, maternal education, maternal employment, and hours per week in daycare, proximal care belief score predicted a self-reported responsive feeding style, *β* = 0.10, *SE* = 0.02, *χ*^2^ = 24.37, *p* < 0.0001 (*β* is the effect estimate, *SE* is the standard error, *χ*^2^ is the chi-squared statistic, and *p* is the calculated probability). In Step 2, physical contact throughout the day and night (via babywearing and co-sleeping) predicted reporting an on-demand feeding philosophy, *β* = 0.62, *SE* = 0.21, *χ*^2^ = 9.13, *p* < 0.001; see Table 3.

We next tested breastfeeding outcomes associated with responsive breastfeeding philosophy with three separate models. Controlling for infant age, maternal education, maternal employment, and hours per week in daycare, responsive feeding philosophy predicted increased likelihood of exclusive breastfeeding (for infants under six months), *β* = 0.50, *SE* = 0.24, *χ*^2^ = 4.33, *p* = 0.04 (Model 1, see Table 4); increased frequency of breastfeeding times per day *β* = 0.84, *SE* = 0.23, *χ*^2^ = 3.64, *p* < 0.001 (Model 2, see Table 4); and longer planned breastfeeding duration, *β* = 2.40, *SE* = 0.70, *χ*^2^ = 3.44, *p* < 0.001 (Model 3, see Table 4).

### 2.3. Discussion

We documented beliefs and practices consistent with proximal care and their relation to self-reported breastfeeding behavior among U.S. mothers. Our first research aim was to assess whether behaviors consistent with proximal care (i.e., mother–infant physical contact throughout the day and night via babywearing and co-sleeping) predicted increased likelihood of reporting a responsive breastfeeding philosophy. Consistent with our predictions, mothers who reported both babywearing and co-sleeping (but not babywearing or co-sleeping only) had an increased likelihood of reporting a responsive feeding philosophy. This finding aligns with ethnographic work showing high levels of breastfeeding responsiveness among populations that practice physical contact throughout the day and night. It is possible that mothers who only practice babywearing or only practice co-sleeping may engage in these practices for convenience, rather than for the desire to have constant physical closeness to infants. This distinction between constant day and night physical contact versus just babywearing or just co-sleeping warrants further investigation.

Our second research aim was to examine whether having a responsive feeding philosophy predicted improved breastfeeding outcomes. Reporting a responsive feeding philosophy predicted increased likelihood of exclusive breastfeeding during the first six months of life, increased feeding frequency, and longer planned breastfeeding duration. The finding regarding planned breastfeeding duration was limited by the fact that this was only in relation to the planned—rather than actual—breastfeeding duration. Future work should employ a longitudinal design to see if responsive feeding does in fact predict actual breastfeeding duration.

One general limitation of this study is that it only reports whether the mother would describe herself as a responsive feeder, which may be closer to her ideal behavior rather than reflecting the mother’s actual behavior at time of feeding. To address this in Study 2, we had mothers fill out a three-day at-home feeding log. At the time of each feeding, mothers documented the reason for feeding their child, as well as their distance from their child (i.e., in physical contact versus not in physical contact) preceding feeding onset, with the aim of capturing a more accurate depiction of the mother’s feeding behavior and how it relates to mother–infant physical contact.

## 3. Study 2

The primary objective of Study 2 was to examine whether individual variation in mother–infant physical contact predicted increased likelihood of feeding in response to early hunger cues (e.g., rooting, lip smacking) rather than waiting for the onset of distress (i.e., crying) or feeding for other reasons (comfort, schedules). Mothers filled out an at-home feeding log for three days. For each feeding, mothers documented the location of the infant (i.e., mother–infant contact) preceding feed onset and the reason for initiating feeding. In line with past ethnographic work citing an association between mother–infant physical contact and increased breastfeeding frequency [42], we predicted increased responsiveness to infant hunger cues when a feeding was preceded by mother–infant physical contact in comparison with mother–infant proximity (without direct physical contact).

Hunger is not the only reason a mother might breastfeed her baby, as feeding may be motivated by mother-led contextual reasons (e.g., work constraints, doctor-recommended schedules, or concerns about breastfeeding in public). Mothers may also feed for infant-led contextual reasons (e.g., use nursing as a strategy for comforting infants, as well as decreasing crying and helping infants get to sleep) [43]. Our second objective was to examine whether increased physical contact predicted increased likelihood of feeding to comfort the infant (as reported by the mother) rather than for adult-led contextual reasons. Based on the proposal that physical contact facilitates mother–infant bonding [44], we predicted that when mothers report feeding for non-hunger reasons, the feeding session would be more likely to be preceded by physical contact when feeding in response to infant-led (i.e., comfort) reasons versus adult-led contextual reasons.

As in Study 1, we tested mothers’ degree of alignment with the beliefs of proximal care culture with Keller’s parental ethnotheory questionnaire. We controlled for these beliefs in all analyses to test whether feeding responsiveness could be attributed to increased mother–infant physical contact, beyond the variation attributed to maternal beliefs. We also tested—and controlled for—the same demographic factors that were included as controls in Study 1 (infant age, maternal education, maternal employment, and hours per week in daycare). These societal factors are some of the primary differences between proximal care and distal care cultures, have been identified as shaping breastfeeding outcomes in the breastfeeding literature, and also may be important because they affect the amount of time a mother spends with her infant.

### 3.1. Materials and Methods

This study was conducted in accordance with the Declaration of Helsinki, and the protocol was approved by the Institutional Review Board of University of California, San Diego (protocol number 130567 “Culture and Infant-Caregiver Interactions”). Study 2 comprised a subset of the participants from Study 1 (recruitment methods and eligibility were identical to Study 1). Only mothers who logged at least 12 breastfeeding sessions over a period of three consecutive days were included in the sample. Because we were only sampling from populations of breastfeeding mothers, the participants in this study are a unique sample and are not representative of U.S. mothers at large.

We used an online questionnaire (Google Forms) to solicit demographic information from each mother, including infant age, maternal age, maternal education, maternal employment (currently working outside of the home versus not), and average hours per week that the infant spends in daycare. Maternal beliefs about responsiveness were assessed with the same questions from Keller’s parental ethnotheory questionnaire that was used in Study 1.

The feeding log consisted of three questions: (1) feed method (breastmilk from breast, breastmilk from bottle, formula in bottle, other liquids, other solids, and other), (2) location of the infant before feeding onset (in physical contact, in visual proximity, and no contact), and (3) reason for feeding (hunger: early cues, hunger: distress, non-hunger: infant-led, and non-hunger: mother-led). The date and time of the feeding session was automatically recorded by the online form. For each of these questions, a list of options was provided and only one response could be chosen for each question. For the question: “Where was your baby when you decided to feed him/her?”, there were three pre-determined mutually exclusive categories of responses with regard to mother–infant contact: (1) physical contact (mother was in direct physical contact with the infant), (2) visual proximity (the mother was near enough to see the infant, but not in physical contact), and (3) no contact (the infant was out of sight or with another caregiver). For the question: “Why did you decide to start feeding your baby?”, there were four pre-determined mutually exclusive categories of responses: (1) hunger: early cues, (2) hunger: distress, (3) non-hunger: infant-led, and (4) non-hunger: mother-led.

Feedings were coded as being in response to cues if the mother indicated that the feeding was initiated because the infant had shown either visual communication (e.g., facial expression), vocal communication (e.g., lip smacking), or physical communication (e.g., breast nuzzling, squirming) that indicated hunger (but not to the point of distress or crying). Feedings were coded as being in response to distress if the mother indicated she had decided to feed because the infant was crying or showing clear distress. Non-hunger feedings were coded as infant-led if the mother initiated feeding for a reason other than hunger that was centered around the well-being of the baby (e.g., wanted to comfort the baby, wanted to calm the baby before getting shots). Non-hunger feedings were coded as mother-led if the mother initiated feeding for a reason other than hunger that was centered around adult-dictated logistical reasons such as schedules (e.g., needing to leave for work) or other maternally-motivated reasons (e.g., breasts feeling engorged).

After indicating interest in the study, mothers were contacted electronically by a research assistant to give details about the feeding log procedure and obtain informed consent. Mothers were instructed to fill out the maternal questionnaire first, then fill out the feeding log during a consecutive three-day period of their choice. Both the questionnaire and the feeding log were administered online via a web browser or smartphone app.

We used generalized mixed-effects logistic regression models to test whether maternal beliefs (proximal care belief score) and immediate physical contact (versus visual contact or no contact) predicted reason for feeding. We analyzed hunger-related reasons for feeding (early cues versus distress) separately from non-hunger reasons for feeding (infant-led versus mother-led). In these models, we controlled for infant age, maternal education, maternal employment, and infant hours in daycare by including these as fixed effects. We included random intercepts for subject, as well as random slopes to account for the multiple responses for each participant [45]. These analyses were conducted using the lme4 package within R Studio software, Version 1.0.44 (RStudio, Inc., Boston, MA, USA) [46].

### 3.2. Results

Ninety-nine breastfeeding mothers completed the feeding log and were included in the final analyses. Infants were 0- to 12-month-olds (51 female, 5.66 months, standard deviation (*SD*) = 3.25). Mothers were 21 to 42 years old (*M* = 30.97 years, *SD* = 4.64) and had completed high school (30.61%), college (38.78%) or a graduate program (30.61%). The average household income of the sample was $78,703 (*SD* = $50,064). Mothers were multiparous (had more than one child, 75.26%) and were exclusively breastfeeding (65.66%). Many of the mothers were not currently working (60.20%), and infant hours in daycare ranged from zero to 55 h per week (*M* = 4.41 h, *SD* = 11.52).

Mothers logged from 12–47 breastfeeding sessions over the course of three days (*M* = 25.86, *SD* = 8.34); see Table 5. An average of 14.15 of the feedings were initiated when the infant was in physical contact with the mother (*SD* = 7.37, 3–39 feeds). The most common reason for feeding was early hunger cues (*M* = 30.52%, *SD* = 16.91%), followed by late cues (*M* = 34.59%, *SD* = 21.53%), infant-led non-hunger reasons (*M* = 17.42%, *SD* = 15.34%), and mother-led non-hunger reasons (*M* = 17.26%, *SD* = 14.33%). Proximal care belief scores ranged from −8 to +17 (*M* = 5.84, *SD* = 5.88) out of a possible range of −20 to +20.

Mother–infant physical contact predicted feeding in response to early hunger cues in comparison with distress, *β* = 0.991, *SE* = 0.315, *z* = 3.149, *p* = 0.002; see Table 6. Mothers who initiated more feedings while in physical contact (i.e., the median 53% or more) had a higher percentage of feeds initiated in response to early cues (*M* = 33.24%, *SE* = 2.41) than mothers who initiated fewer feedings (less than 53%) while in physical contact (*M* = 25.67%, *SE* = 2.59). Visual contact did not predict feeding in response to early cues versus distress, *β* = 0.002, *SE* = 0.200, *z* = 0.007, *p* = 0.994.

For non-hunger feedings, the bivariate regression analysis revealed that physical contact predicted feeding for infant-led versus mother-led reasons, *β* = 1.271, *SE* = 0.261, *z* = 4.868, *p* < 0.0001. Controlling for demographic factors and multiple responses (i.e., feeding log entries), we found that physical contact predicted feeding for infant-led versus mother-led reasons, *β* = 1.246, *SE* = 0.304, *z* = 4.095, *p* < 0.0001; see Table 7. Mothers with more feedings initiated in physical contact had a lower percentage of feeds initiated for mother-led reasons (*M* = 14.54%, *SE* = 1.94) than mothers with fewer feedings initiated in physical contact (*M* = 22.22%, *SE* = 2.08). In contrast, visual contact did not predict feeding for infant-led versus mother-led reasons, *β* = 0.397, *SE* = 0.288, *z* = 1.379, *p* = 0.168.

### 3.3. Discussion

These data provide support for the proposal that mother–infant physical contact influences maternal responsiveness to early hunger cues during breastfeeding. Consistent with our predictions, mothers were more likely to respond to early hunger cues when in physical contact with their infant. Visual contact did not predict reason for feeding, suggesting that it is something unique about physical contact that facilitates increased maternal responsiveness. In addition, increased responsiveness to hunger cues was not simply attributed to increased feeding frequency overall.

We tested whether physical contact predicted infant-led non-hunger reasons for feeding. When feeding for non-hunger contextual reasons, mothers were more likely to feed for infant-led reasons (e.g., to comfort the infant) rather than adult-led reasons (e.g., schedules) if the feeding was preceded by mother–infant physical contact. Visual contact was not associated with feeding for non-hunger reasons, suggesting that there is something special about direct physical contact that facilitates infant-led motivations for feeding above and beyond just having the infant in proximity.

In addition to testing specific research questions, we also sought to document proximal care parenting practices among Euro-American middle class parents. Though caregiving in U.S. culture is typically characterized as distal care; the mothers in our sample participated in many parenting practices typical of proximal care culture, including babywearing and co-sleeping. This study provides insight into how proximal care practices might shape other components of infant-caregiver interaction during feeding. More research is needed to examine the implications of these practices for infant health and nutrition.

## 4. General Discussion

Convergent methods were used to test whether mother–infant physical contact predicts increased responsiveness to early hunger cues during breastfeeding. Both the self-report questionnaire and the at-home feeding log showed that maternal beliefs and practices characteristic of proximal care culture predicted increased maternal responsiveness to infant hunger cues during breastfeeding. We discuss potential mechanisms underlying the connection between physical contact and maternal responsiveness, present potential directions for future research, and discuss broader implications of this work for protecting and promoting breastfeeding.

### 4.1. Mechanisms Underlying the Effect of Physical Contact

Our data show that mother–infant physical contact predicts increased responsiveness to infant hunger cues during breastfeeding, beyond any variation explained by underlying beliefs about responsiveness. Thus, mother-infant physical contact may facilitate increased maternal awareness of her infant’s emotional state and communicative intentions, allowing her to increase her responsiveness to subtle movements or physiological changes in the infant that cannot be observed but can be felt. The release of oxytocin—a neuropeptide involved in mammalian social bonds—during mother–infant physical contact may also underlie the effect of physical contact on maternal responsiveness. Oxytocin is associated with some aspects of responsiveness, including responding to infant crying [47] and infant laughing [48]. Because oxytocin is released during skin-to-skin contact [49], infant holding without direct skin-to-skin [50], and even in response to infant vocalizations [51], oxytocin release likely plays a role in the relationship between mother–infant physical contact and maternal responsiveness demonstrated in these studies. Though measuring oxytocin was outside of the scope of the current project, future research should measure the effect of mother–infant contact on maternal responsiveness while accounting for potential changes in oxytocin levels.

### 4.2. Limitations and Broader Implications

Because we did not directly manipulate mother–infant physical contact in these studies, we cannot determine the causal relationship between mother–infant physical contact and responsive feeding. We hope these studies motivate controlled experimental studies to continue investigating our hypothesis that mother–infant physical contact facilitates increased responsiveness to infant hunger cues during breastfeeding. Because we specifically sampled from the population of breastfeeding mothers, mothers in our study were more likely to be older, more educated, and have a higher income than the general population. We therefore cannot determine whether these findings generalize to the population of U.S. mothers at large. Many studies have reported that crying is the most commonly reported indication of hunger used by U.S. mothers to initiate feeding [52]. The fact that mothers in this sample initiated feeding more often due to early hunger cues rather than distress in Study 2 demonstrates higher levels of responsiveness than the average population. Past research shows that breastfeeding mothers show different patterns of interaction and responsiveness than bottle-feeding mothers. Breastfeeding mothers—in comparison with bottle-feeding mothers—are more likely to show an increase in oxytocin levels after holding their infant [53] and are more likely to show neural activation in response to their infant’s cry [54], suggesting that the variation in responsiveness found in these studies may be specific to breastfeeding mothers.

Populations show substantial variation in the modality of infant-caregiver interaction, especially with regard to physical contact [55]. The amount of physical contact with infants in Western, educated, industrialized, rich, and democratic—“WEIRD” societies, which comprise the majority of research on infant nutrition and development [56,57]—is substantially lower than in many other human populations [58,59]. Infant care in WEIRD societies is increasingly dominated by products that limit physical contact between infants and caregivers (e.g., cribs, strollers, playpens, and bouncers). Because human infants are like all other primates in their need to maintain close contact with mothers, this lack of physical contact represents a caregiving method that is unique from a cultural and historical perspective [60]. Though past research has identified the importance of cultural ecologies on breastfeeding behavior—including both cultural beliefs and behaviors [61]—our data suggest that amount of physical contact with infants may shape breastfeeding behavior, presenting a new avenue for exploring the intersection between social interaction and early nutrition.

Skin-to-skin contact is beneficial for physiological stability, physical growth, and breastfeeding initiation for both preterm and full-term infants, yet investigating the implications of mother–infant physical contact for responsiveness to hunger cues during breastfeeding is surprisingly understudied. Randomized controlled trials with preterm infants show that skin-to-skin contact immediately after birth increases the likelihood of breastfeeding in the hospital and throughout the first postpartum months [62], while also leading to a more stable heartbeat, respiratory rate, body temperature, and other benefits [63]. Intervention studies with full-term infants show that increased physical contact through carrying facilitates more secure attachment [64] and increased frequency of breastfeeding [65]. There are still substantial gaps in our knowledge of the processes underlying the effects of skin-to-skin and physical contact, leaving many questions unanswered about how and why physical contact can be used to improve breastfeeding outcomes for infants. Because increasing mother–infant physical contact is both a viable and inexpensive potential intervention, this area warrants further research.

### 4.3. Conclusions

Breastmilk is internationally recognized as the optimal nutrition for infant health and development. Neglecting to recognize and respond to subtle feeding cues exacerbates both physiological and psychological breastfeeding challenges, yet many mothers feed on a schedule or report crying rather than early hunger cues as the primary motivation for initiating feeding. Our data suggest that culturally-mediated parenting practices like mother–infant physical contact may shape maternal responsiveness to early hunger cues, providing a new potential opportunity for intervention to support breastfeeding mothers in meeting their goals.

## Figures and Tables

**Table 1:** Demographic information for the participants in Study 1.

Maternal and Infant Characteristics	Range	*M*	*SD*
Infant Age	0.23–24.91	9.36	5.92
Maternal Age	20–44	30.71	(4.29)
Daycare	0–60	9.70	(15.59)
		*n*	%
**Maternal Education**			
High School		174	30.16%
College Degree		204	35.36%
Graduate Degree		199	34.49%
**Maternal Employment**			
Home		345	55.11%
Working		281	44.89%

*M* is the mean response of each category; *SD* is the standard deviation of each category; *n* is number of caregivers in the sample who fit into each category; percentages provided are based on the total sample. Infant age was measured in months, maternal age was measured in years, and daycare was measured as hours per week that the child spends in daycare.

**Table 2:** Descriptive statistics for the participants in Study 1.

Maternal Characteristics	*n*	%
**Feeding Philosophy**		
Responsive Feeding	441	71.13%
Other (Schedule/Mixed)	179	28.87%
**Co-sleeping**		
Yes	266	42.42%
No	361	57.58%
**Babywearing**		
Yes	439	73.41%
No	159	26.59%
**Exclusive Breastfeeding** (for infants 6 months and younger, *n* = 217)		
Yes	177	81.94%
No	39	18.06%
	***M***	***SD***
**Maternal Beliefs**		
Proximal Care Belief Score	6.85	5.73
**Breastfeeding Duration**	21.55	11.76
**Breastfeeding Frequency**	6.92	4.33

*M* is the mean response of each category; *SD* is the standard deviation of each category; *n* is number of participants in the sample.

**Table 3:** Results of the logistic regression predicting responsive breastfeeding philosophy from proximal care practices and beliefs in Study 1.

	Step 1						Step 2					
**Multivariate Analyses**	*β*	*SE*	*χ^2^*	*p*	*Lower 95%*	*Upper 95%*	*β*	*SE*	*χ^2^*	*p*	*Lower 95%*	*Upper 95%*
**Intercept**	1.32	0.24	30.37	<0.0001	0.86	1.80	1.38	0.27	26.94	<0.0001	0.87	1.92
**Infant Age**	−0.07	0.02	15.18	<0.0001	−0.11	−0.04	−0.07	0.02	15.20	<0.0001	−0.11	−0.04
**Maternal Education**												
**High School (ref)**												
**College**	−0.03	0.15	0.04	0.85	−0.32	0.27	−0.07	0.15	0.20	0.65	−0.37	0.23
**Graduate**	−0.17	0.15	1.32	0.25	−0.47	0.12	−0.10	0.15	0.41	0.52	−0.40	0.20
**Maternal Employment**												
**Home (ref)**												
**Working**	0.33	0.14	5.68	0.02	0.06	0.60	0.34	0.14	5.84	0.02	0.07	0.61
**Daycare**	−0.02	0.01	7.52	0.01	−0.04	−0.01	−0.02	0.01	6.50	0.01	−0.04	−0.01
**Maternal Beliefs**												
**Proximal Care Belief Score**	0.10	0.02	24.37	<0.0001	0.06	0.14	0.08	0.02	12.06	<0.001	0.03	0.12
**Mother-Infant Physical Contact**												
**Neither (ref)**												
**Babywearing Only**							0.01	0.18	0.00	0.97	−0.34	0.35
**Co-sleeping Only**							−0.16	0.30	0.27	0.60	−0.73	0.46
**Babywearing and Co-sleeping**							0.62	0.21	9.13	<0.001	0.22	1.03

*β* is the effect estimate; *SE* is the standard error; *χ*^2^ is the chi-squared statistic; *p* is the calculated probability; *lower 95%* is the lower bounds of the 95% confidence interval; *upper 95%* is the upper bounds of the 95% confidence interval.

**Table 4:** Results of the logistic regression predicting exclusive breastfeeding (feeding only breastmilk to infants under six months) from self-reported responsive feeding (Model 1), results of the linear regression predicting feeding frequency (average number of breastfeeding sessions per day) from self-reported responsive feeding (Model 2), and results of the linear regression predicting planned breastfeeding duration (in months) from self-reported responsive feeding (Model 3) in Study 1.

**Model 1: Exclusive Breastfeeding**	***β***	***SE***	***χ*^2^**	***p***	***Lower 95%***	***Upper 95%***
Intercept	2.19	0.54	16.41	<0.0001	1.18	3.32
**Infant Age**	−0.27	0.12	4.78	0.03	−0.53	−0.03
**Maternal Education**						
High School (ref)						
College	0.10	0.32	0.10	0.75	−0.50	0.75
Graduate	−0.30	0.30	1.00	0.32	−0.89	0.30
**Maternal Employment**						
Home (ref)						
Working	0.11	0.25	0.20	0.65	−0.37	0.63
**Daycare**	0.01	0.02	0.37	0.54	−0.02	0.05
**Feeding Philosophy**						
Schedule/Other (ref)						
Responsive Feeding	0.50	0.24	4.33	0.04	0.02	0.97
**Model 2: Breastfeeding Frequency**	***β***	***SE***	***χ*^2^**	***p***	***Lower 95%***	***Upper 95%***
Intercept	8.58	0.43	19.72	<0.0001	7.73	9.43
**Infant Age**	−0.18	0.03	−5.36	<0.0001	−0.25	−0.12
**Maternal Education**						
High School (ref)						
College	0.24	0.30	0.80	0.42	−0.34	0.82
Graduate	−0.26	0.29	−0.91	0.36	−0.82	0.30
**Maternal Employment**						
Home (ref)						
Working	−0.29	0.25	−1.15	0.25	−0.77	0.20
**Daycare**	−0.03	0.02	−1.85	0.07	−0.06	0.00
**Feeding Philosophy**						
Schedule/Other (ref)						
Responsive Feeding	0.84	0.23	3.64	<0.001	0.39	1.29
**Model 3: Breastfeeding Duration**	***β***	***SE***	***χ*^2^**	***p***	***Lower 95%***	***Upper 95%***
Intercept	15.75	1.41	11.17	<0.0001	12.98	18.52
**Infant Age**	0.45	0.11	4.15	<0.0001	0.24	0.67
**Maternal Education**						
High School (ref)						
College	−1.80	0.94	−1.92	0.06	−3.64	0.05
Graduate	1.11	0.86	1.28	0.20	−0.59	2.81
**Maternal Employment**						
Home (ref)						
Working	−2.60	0.82	−3.19	<0.001	−4.21	−0.99
**Daycare**	0.00	0.05	0.04	0.97	−0.10	0.10
**Feeding Philosophy**						
Schedule/Other (ref)						
Responsive Feeding	2.40	0.70	3.44	<0.001	1.03	3.77

Exclusive breastfeeding was defined as feeding only breastmilk to infants and this model only included a sub-sample of infants under six months of age (*n* = 217); breastfeeding frequency was defined as the average number of breastfeeding sessions per day; breastfeeding duration was the planned number of months of breastfeeding.

**Table 5:** Descriptive statistics for Study 2.

Feeding and Infant Care Characteristics	Range	*M*	*SD*
**Total Breastfeeding Sessions**	12–47	23.63	7.77
**Initiated in Physical Contact**	3–39	13.07	6.74
**Initiated in Visual Contact**	0–24	7.85	4.54
**Hunger—Early Cues**	0–23	6.06	4.19
**Hunger—Distress**	2–22	10.85	5.45
**Non-Hunger—Infant-Led**	0–9	3.02	2.79
**Non-Hunger—Mother-Led**	0–9	3.05	2.62
		*n*	%
**Exclusive Breastfeeding for 6 Months**			
**Yes**		35	85.37%
**No**		6	14.63%
**Babywearing**			
**Yes**		29	70.73%
**No**		12	29.27%
**Co-sleeping**			
**Yes**		17	41.46%
**No**		24	58.54%

*M* is the mean response of each category; *SD* is the standard deviation of each category; *n* is number of participants in the sample.

**Table 6:** Model Predicting Responsiveness to Cues. Fixed effects for the mixed-effects model predicting initiating hunger-related feedings in response to early cues (in comparison with crying) in Study 2.

Multivariate Analyses	*β*	*SE*	*z*	*p*
**Infant Age**	−0.030	0.044	−0.665	0.506
**Maternal Education**				
**High School (ref)**				
**College**	−0.081	0.343	−0.235	0.814
**Graduate**	0.740	0.390	1.898	0.058
**Maternal Employment**				
**Home (ref)**				
**Working**	−0.604	0.326	−1.853	0.064
**Daycare**	0.044	0.016	2.844	0.004
**Maternal Beliefs**				
**Proximal Care Belief Score**	0.030	0.027	1.106	0.269
**Mother–Infant Physical Contact**				
**No Contact (ref)**				
**Visual Contact**	0.002	0.300	0.007	0.994
**Physical Contact**	0.991	0.315	3.149	0.002

*β* is the effect estimate; *SE* is the standard error; *z* is the z-score; *p* is the calculated probability.

**Table 7:** Model Predicting Responsiveness to Cues. Fixed effects for the mixed-effects model predicting initiating non-hunger feedings in response to infant comfort (in comparison with adult-determined reasons) in Study 2.

Multivariate Analyses	*β*	*SE*	*z*	*p*
**Infant Age**	0.067	0.044	1.514	0.130
**Maternal Education**				
**High School (ref)**				
**College**	0.027	0.345	0.079	0.937
**Graduate**	0.085	0.403	0.210	0.833
**Maternal Employment**				
**Home (ref)**				
**Working**	−0.446	0.319	−1.397	0.162
**Daycare**	−0.013	0.015	−0.897	0.369
**Maternal Beliefs**				
**Proximal Care Belief Score**	0.057	0.022	2.665	0.008
**Mother–Infant Physical Contact**				
**No Contact (ref)**				
**Visual Contact**	0.397	0.288	1.379	0.168
**Physical Contact**	1.246	0.304	4.095	<0.0001
